# Erlotinib as single agent first line treatment in locally advanced or metastatic activating *EGFR* mutation-positive lung adenocarcinoma (CEETAC): an open-label, non-randomized, multicenter, phase IV clinical trial

**DOI:** 10.1186/s12885-018-4283-z

**Published:** 2018-05-25

**Authors:** Zsolt Markóczy, Veronika Sárosi, Iveta Kudaba, Gabriella Gálffy, Ülkü Yilmaz Turay, Ahmet Demirkazik, Gunta Purkalne, Attila Somfay, Zsolt Pápai-Székely, Erzsébet Rásó, Gyula Ostoros

**Affiliations:** 1 National Koranyi Institute of TB and Pulmonology, Piheno ut 1, Budapest, H-1122 Hungary; 2 Division of Pulmonology, University of Pecs, Pecs, Hungary; 3 Riga East University Hospital Oncology Center, Riga, Latvia; 4 Department of Pulmonology, Semmelweis University, Budapest, Hungary; 5 Clinic of Chest Diseases, Ataturk Chest Diseases and Chest Surgery Training and Research Hospital, Ankara, Turkey; 6 Department of Medical Oncology, Ibn-i Sina Hospital, Ankara University Medical Faculty, Ankara, Turkey; 7 Oncology Institute, Riga Stradins University, Riga, Latvia; 8 Department of Pulmonology, University of Szeged, Szeged, Hungary; 9 St. George Hospital of Fejer County, Szekesfehervar, Hungary; 10 2nd Department of Pathology, Semmelweis University, Budapest, Hungary

**Keywords:** Non-small cell lung cancer, Lung adenocarcinoma, EGFR, Erlotinib

## Abstract

**Background:**

Erlotinib is approved for the first line treatment of epidermal growth factor receptor (EGFR) mutation-positive non-small cell lung cancer. Since the number of prospective studies in Caucasian patients treated in routine clinical setting is limited we conducted a multicenter, phase IV clinical trial to determine the efficacy and safety of erlotinib and to demonstrate the feasibility of the validated standardized companion diagnostic method of EGFR mutation detection.

**Methods:**

651 chemonaive, cytologically or histologically verified advanced stage lung adenocarcinoma patients from Hungary, Turkey and Latvia were screened for exon19 microdeletions and exon21 L858R EGFR mutations using the companion diagnostic EGFR test. EGFR mutation-positive, locally advanced or metastatic lung adenocarcinoma patients received as first line treatment erlotinib at 150 mg/day. The primary endpoint was progression-free survival (PFS).

**Results:**

62 EGFR mutation-positive patients (9.5% of screened) were included in the safety/intent-to-treat cohort. Median PFS was 12.8 months (95%CI, 9.9–15.8), objective response rate and one-year survival was 66.1% and 82.5%, respectively. Most frequent treatment related adverse events were diarrhoea and rash. Eastern Oncology Cooperative Group Performance Status (ECOG PS), smoking status and M1a/M1b disease stage were significant prognosticators of PFS (*p* = 0.017, *p* = 0.045 and *p* = 0.002, respectively). There was no significant difference in PFS between the subgroups stratified by gender, age or exon19 vs exon21 mutation.

**Conclusions:**

Our study confirmed the efficacy and safety of first line erlotinib monotherapy in Caucasian patients with locally advanced or metastatic lung adenocarcinoma carrying activating EGFR mutations based on the screening with the approved companion diagnostic procedure***.***

**Trial registration:**

ClinicalTrials.gov Identifier: NCT01609543.

## Background

Lung cancer is the leading cause of cancer related mortality worldwide [1]. In European lung adenocarcinoma patients, the incidence of mutations of epidermal growth factor receptor (EGFR) gene is between 5 to 10% [2–4]. Currently, three EGFR tyrosine kinase inhibitors (EGFR-TKIs) including the first-generation, reversible TKI erlotinib and gefitinib as well as the second-generation irreversible TKI afatinib are approved for the treatment of patients with locally advanced or metastatic non-small cell lung cancer (NSCLC) with EGFR activating mutations [5]. For all three agents, the classic mutations of L858R and exon 19 microdeletions can serve as positive predictive biomarkers for response. Of note, a number of additional so called rare EGFR mutations are also sensitizing for EGFR-TKI therapy [4]. Erlotinib was demonstrated to delay symptom progression, improve quality of life and prolong survival as a first-line treatment when compared to standard chemotherapy in patients with exon 19 deletions or exon 21 (L858R) substitution mutations of EGFR [6, 7]. In addition, erlotinib is also approved by the European Medicines Agency for the treatment of patients with locally advanced or metastatic NSCLC after failure of at least one prior chemotherapy regimen (with no mutation analysis requirement) as well as for switch maintenance treatment in patients with locally advanced or metastatic NSCLC with EGFR activating mutations and stable disease after first-line chemotherapy [8].

EGFR mutations are associated with adenocarcinoma histology and more often found in non-smokers. Especially in Asian populations it is also more frequent in females often associates with younger age [9]. These epidemiological characteristics often influence the screening strategy. The presence of KRAS mutations is in general mutually exclusive with EGFR mutations and associates with the lack of response to EGFR-TKIs [10–12].

Our multicenter, phase IV clinical trial was designed to determine the efficacy and safety of erlotinib in routine clinical practice and to demonstrate the feasibility of the validated standardized companion diagnostic method of EGFR mutation detection in Caucasian patients.

## Patients and methods

### Patients

The CEETAC (ClinicalTrials.gov Identifier: NCT01609543) open-label, non-randomized, multicenter trial investigated the efficacy and safety of first line erlotinib monotherapy in routine clinical practice in 10 Hungarian, 5 Turkish and 2 Latvian clinical centers. 651 chemonaive, inoperable, advanced stage lung adenocarcinoma patients were screened for EGFR mutation. Patients above the age of 18 years with histologically or cytologically verified, inoperable, locally advanced, recurrent or metastatic lung adenocarcinoma carrying an activating EGFR mutation (exon 19 microdeletions or exon 21 L858R point mutation) by using Cobas® 4800 EGFR Mutation Test at a designated central laboratory were included in the safety as well as in the intent-to-treat cohort. 35 Hungarian, 15 Turkish and 12 Latvian patients were eligible to participate in the study. All participants had to have an Eastern Cooperative Oncology Group Performance Status (ECOG PS) between 0 and 2 and a life expectancy of at least 12 weeks. All patients provided written informed consent.

### Mutation analysis

Formalin-fixed paraffin-embedded histological specimens or stained cytological samples were assessed by pathologists at light microscopy and tumour-rich areas were macrodissected in sections and tumor to normal ratio was determined. Cobas® DNA Sample Preparation Kit (Roche Molecular Diagnostics) was then used to isolate DNA according to the manufacturer’s instructions. The purity and the concentration of the extracted DNA was determined by Nanodrop 2000 (Thermo Fisher Scientific).

EGFR mutation analysis was carried out by Cobas® EGFR Mutation Test (Roche Molecular Diagnostics), a real-time PCR test for the qualitative detection of mutations for which the safety and efficacy of erlotinib use have been established: exon 19 deletions and exon 21 substitution L858R. The measurement was carried out by Cobas® z 480 analyzer (Roche Molecular Diagnostics) according to the manufacturer’s instructions using the Cobas® 4800 System Microwell Plate and the primers, probes and internal controls supplied with the kit.

### Treatment and follow-up

Patients received erlotinib 150 mg/day orally until disease progression, withdrawal of consent or intolerable adverse events. Examination of vital signs and routine hematology was performed at every 28-day visit. Tumor measurement and response grading was performed according to institutional standard of care in line with RECIST version 1.1 and with a maximum interval of 2 months. The primary endpoint was progression-free survival (PFS), secondary endpoints were objective tumor response, one-year survival and safety. PFS was defined from the start of erlotinib treatment to the first documented progression or death. Patients without progression were censored on the date of last evaluable tumor assessment.

### Statistical analysis

The association of gender, disease stage and smoking with the exon19 and exon 21 subgroups was tested with Fisher’s exact test. The difference in age and ECOG score in these subgroups was tested by unpaired t-test and by Chi-square test, respectively. PFS was estimated with the Kaplan-Meier method and described with the median value and two-sided 95% confidence interval (CI). Exploratory subgroup analyses of progression-free survival were done with two-sided log-rank test and the ratio of the median survival times including its 95% confidence intervals are also reported. Multivariate analysis was performed using the Cox-regression model to calculate hazard ratios (HRs) and corresponding confidence intervals. Statistical differences with *p* < 0.05 were considered significant.

## Results

### Patient characteristics

**Fig. 1:**
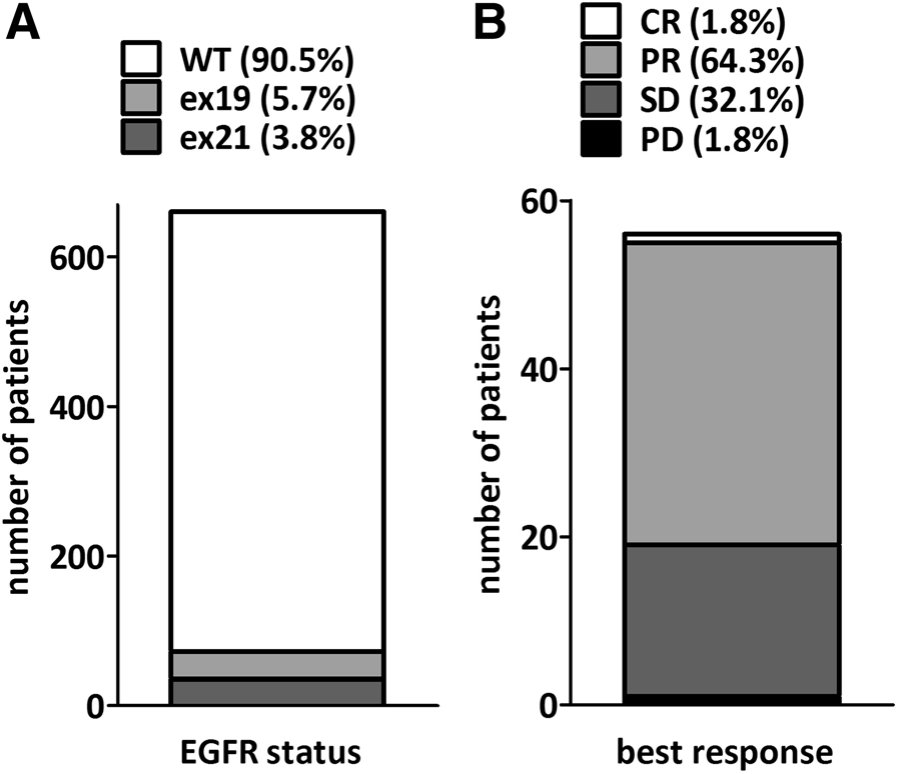
EGFR mutation status in the screened population and best response to first line erlotinib treatment. **(a)** Out of the 651 screened patients, 62 carried EGFR mutations including 37 in exon 19 and 35 in exon 21. **(b)** The distribution of best response among the 56 intent-to-treat patients. (CR – complete remission, PR – partial response, SD – stable disease, PD – progressive disease)

Out of the 651 screened patients, 62 with EGFR mutant lung adenocarcinoma were enrolled from 17 participating centers between March 2012 and January 2014 (Fig. 1A). The last study visit was in January 2015 and data collection was closed in June 2015. The clinicopathological characteristics dichotomized by exon 19 and 21 mutations are summarized in Table 1. All but two patients were of Caucasian origin. Mean age at the time of enrollment was 70.5 (range 28 to 86) years. There were 50 female and 12 male patients included. 6 patients (9.7%) were enrolled with stage IIIb disease. Among the 56 stage IV cases, the most frequently affected sites included 45 (80.4%) intrapulmonary, 19 (33.9%) bone and 7 (12.5%) liver metastasis at the time of enrollment.

**Table 1:** Clinicopathological characteristics of the 62 patients dichotomized by exon19 microdeletions and exon21 mutations

		Total	Exon19	Exon21	*p*-value
Number of patients		62 (100%)	37 (66.1%)	25 (33.9%)	
Age (median, range)		70.5 (28–86)	67 (28–81)	71 (48–86)	0.088
Gender	Male	12 (19.4%)	8 (21.6%)	4 (16%)	0.747
	Female	50 (80.6%)	29 (78.4%)	21 (84%)	
ECOG performance status	0	24 (38.7%)	17(46%)	7(28%)	0.182
	1	32 (51.6%)	17(46%)	15(60%)	
	2	6(9.7%)	3(8%)	3 (12%)	
Smoking status	Never-smoker	42(67.7%)	25 (67.5%)	17 (68%)	1.00
	Ever smoker	20(32.3%)	12 (32.5%)	8 (32%)	
Tumor Stage	IIIb	6 (9.7%)	1 (1.6%)	5(20%)	0.035
	IV	56 (90.3%)	36(98.4%)	20(80%)	

Data shown in parentheses are column percentages;

### Treatment safety

During the study, 418 adverse events were recorded from 56 patients. 88.2% of adverse events were grade I and II. 159 non-serious adverse events (38%) and 15 serious adverse events (3.6%) were reported to have causal relationship with erlotinib treatment. The list of non-serious adverse events that occurred at a frequency of 5% or more is presented in Table 2. The most frequent adverse events were diarrhoea and rash. Due to adverse events dose modification was applied in fifteen patients (24.1%) and erlotinib withdrawal was necessary in 5 (8.1%) cases.

**Table 2:** Non-serious adverse events during erlotinib treatment

Adverse event	Number of patients (%)	Number of events
Rash	36 (58.1%)	43
Diarrhoea	17 (27.4%)	26
Dry skin	11 (17.7%)	15
Pruritus	9 (14.5%)	10
Asthenia	5 (8.1%)	9
Conjunctivitis	6 (9.7%)	8
Back pain	7 (11.3%)	8
Cough	8 (12.9%)	8
Alopecia	7 (11.3%)	7
Anaemia	5 (8.1%)	6
Growth of eyelashes	5 (8.1%)	5
Nausea	4 (6.5%)	5
Increased blood AP	5 (8.1%)	5
Weight loss	4 (6.5%)	5
Headache	5 (8.1%)	5
Dyspnoea	5 (8.1%)	5
Decreased appetite	4 (6.5%)	4

### Treatment efficacy

**Fig. 2:**
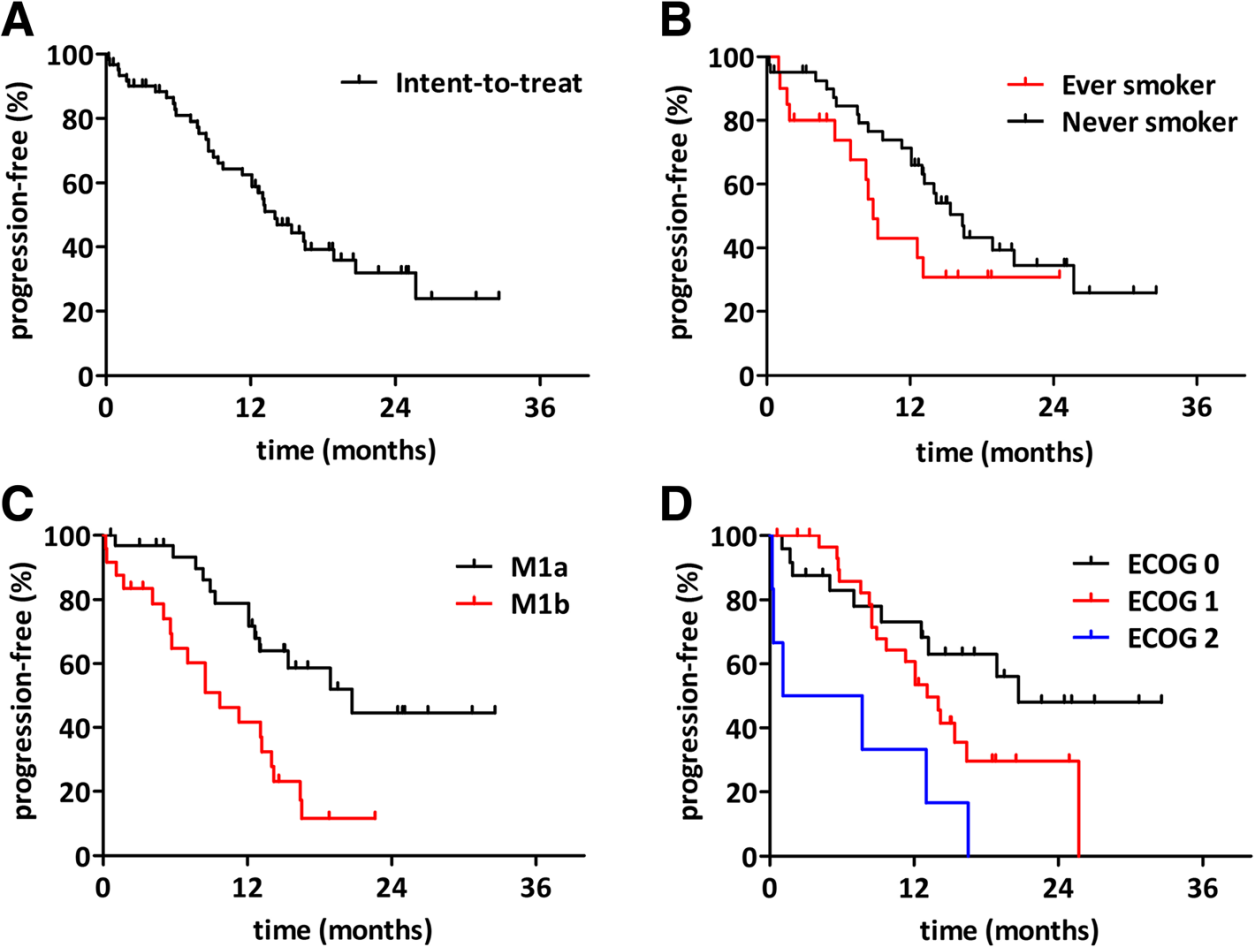
Kaplan-Meier analysis of progression-free survival. **(a)** The intent-to-treat cohort had a 12.8 months median PFS. **(b)** Never smoker patients had increased progression-free survival. **(c)** Patients with extrathoracic metastasis at the time of screening had lower PFS than patients with pulmonary metastasis. **(d)** ECOG2 patients demonstrated progression earlier when compared to ECOG1 or ECOG0 performance status

Best overall response rate could be established for 56 patients (90.3% of the intent-to-treat population). One (1.8%) complete and 36 (64.3%) partial responses as well as 18 stable (32.1%) disease were registered at response evaluation resulting in a disease control rate of 98.2% (Fig. 1B). Progressive disease (PD) as best tumor response was reported in one patient (1.8%). 28 patients (45.2%) discontinued treatment due to disease progression. 11 disease progression related death occurred during the study. Accordingly, the number of events in the PFS analysis was 40. The median follow-up time was 13.4 months (range 1.3 to 32.9). The progression-free survival was 12.8 months (95% CI, 9.9–15.8; Fig. 2A).

### Exploratory subgroup analysis for progression-free survival

The progression-free survival in the various subgroups was compared in order to identify the clinicopathological variables that influence treatment outcome (Table 3). There was no significant difference in PFS between the subgroups stratified by gender, age (using the median 70 years of age as cutoff) or mutation exon 19 or 21. In contrast, ECOG PS was a significant predictor of progression-free survival (*p* = 0.017; Fig. 2D). Never smoker patients also had a significantly increased PFS when compared to ever-smokers (15.4 versus 8.4 months; *p* = 0.045; Fig. 2B). Furthermore, M1a disease at the time of screening also associated with increased progression-free survival when compared to M1b (18.9 versus 8.5 months; *p* = 0.002; Fig. 2C). Finally, we performed multivariate analyses using a multimodality Cox-regression model adjusted for smoking, ECOG and M stage. After multivariate PFS analyses, smoking (HR: 2.07, CI: 1.04–4.14, *p* = 0.039) and ECOG (HR: 2.21, CI: 1.21–4.04, p = 0.01) remained significant factors while M stage (HR: 0.51, CI: 0.25–1.06, *p* = 0.07) did not reach significance.

**Table 3:** Exploratory subgroup analysis of progression-free survival (*n* = 61)

		*N*	PFS (months)	Ratio (CI)	*P*	HR (CI)
Gender	Female	49	13.2	1.56 (1.101–2.023)	0.447	0.719 (0.305–1.696)
	Male	12	8.45			
Age	≤70	31	11.7	0.76 (0.225–1.294)	0.164	1.561 (0.834–2.922)
	> 70	30	15.4			
Smoking	Never	41	15.4	0.545 (0.018–1.073)	0.045	2.118 (1.016–4.418)
	Ever	20	8.4			
Mutation	Exon 19	37	13	0.79 (0.266–1.320)	0.607	1.186 (0.627–2.243)
	Exon 21	24	16.4			
ECOG	0	24	18.9		0.017	
	1	31	12.6			
	2	6	4.4			
Stage^a^	M1a	31	18.9	2.22 (1.708–2.739)	0.002	0.334 (0.167–0.672)
	M1b	24	8.5			

^a^6 IIIb stage patients were excluded from this analysis

## Discussion

In contrast to Asian lung adenocarcinoma patients, there is relatively less information about the first-line EGFR-TKI treatment response rates and progression-free survival in Caucasian cohorts. Our non-randomized, multicenter, phase IV clinical trial including first-line erlotinib treated lung adenocarcinoma patients with activating EGFR mutations was conducted for the very reason. The ORR in our study is in accordance with studies from East-Asia where RRs were found to be 70–75% [13, 14]. Interestingly, the LUX-Lung 2 phase II trial of the second-generation covalent TKI inhibitor afatinib demonstrated similar RR in classic EGFR mutant patients [15]. Of note, the 12.8-months median PFS in our cohort is in the range of previously published data (9.4–11.9 months) from other studies [14, 16].

Previously, three major randomised, phase III first-line studies have shown a tendency for improved response to erlotinib in patients with exon19 microdeletion-positive tumors when compared to L858R-positive cases [6, 7, 17]. This tendency has not been observed in the current study, however, the number of patients is limiting the identification of smaller differences in the progression-free survival. Of note, there was no difference at all in the smoking status of patients in the exon 19 and exon 21 mutation subgroup.

While smoking is clearly the most important factor in the development of lung cancer, the prognostic and predicitve significance of smoking status is context dependent. In several studies, never-smokers have improved OS, however, the increased survival is at least in part due to the overall better performance score and the lack of smoking related comorbidities [18–21]. In standard chemotherapy regimens, predictive value of smoking status is limited [19, 22, 23] or only slightly increased survival can be demonstrated in never-smokers when compared to smokers [24, 25]. In contrast, never-smokers seem to have survival benefits in cohorts that include EGFR-TKI treated patients [4]. Owing to the fact that mutation of EGFR gene is more frequent in non-smokers [26, 27], our study was biased towards never-smokers. Of note, in the ever smoker group there are 4 patients out of 6 are censored beyond one-year that might suggest a decreasing relative risk over time. Nevertheless, there are only 20 patients in the ever smoker subcohort including 3 current smokers. Thus the size of the subcohort prevent us from studying the time-dependent impact of smoking on the progression–free survival. Nevertheless, we found a significantly improved progression-free survival in never-smokers. One reason might be the smoking induced additional mutational load that can lead to the presence of tumor cell clones with resistance mutations and thus lead to earlier emergence of therapy resistance.

With regards to other clinicopathological parameters, we found no significant difference between the gender-specific PFS. However, the patient population in the present study was biased towards female gender and thus this analysis should be interpreted rather carefully due to the low number (*n* = 12) of male patients in the study.

In the present patient cohort ECOG PS was a significant predictor of PFS. It is in line with a number of other studies where better performance status associated with improved anti-EGFR therapy response [4, 28].

Other studies have previously demonstrated that disseminated disease stage have an impact of progession-free survival in EGFR-TKI treated patients [29]. In the current study, there were only 6 non-metastatic patients included, thus the exploratory analysis of stage as a predictor was limited to M1a and M1b subgroups. Of note, patients with metastasis limited to the lung and pleural or pericardial effusion had a signficantly longer PFS when compared to patients with distant organ dissemination.

## Conclusion

Overall, our phase IV clinical trial demonstrated that the companion diagnostic method could identify lung adenocarcinoma patients with activating EGFR mutation with a frequency that was found in other Caucasian patient populations. Furthermore, it has been demostrated that the first-line erlotinib treatment of this patient cohort had an efficacy and safety profile similar to previous large randomized phase III clinical trials.

## Abbreviations:

CI: confidence interval
CR: complete remission.
ECOG: PS Eastern Oncology Cooperative Group performance status.
PD: progressive disease.
PFS: Progression-free survival.
PR: partial response.
RR: response rate.
SD: stable disease.
